# Supplementary material for: Patterns of Intron Gain and Loss in Fungi
Source: PLoS Biol. 2004 Nov 30;2(12):e422. doi: 10.1371/journal.pbio.0020422 (PMC532390; doi:10.1371/journal.pbio.0020422)
Supplement: Table S1 — Also available at http://genes.mit.edu/NielsenEtAl/. (4.3 MB ZIP). [file pbio.0020422.st001.zip › NielsenEtAl/html/884.html]

AN2216.1.NCU07000.1.MG01436.1.FG09604.1


```
 CLUSTAL W (1.82) Multiple Sequence Alignments - Introns Inserted


Sequence 1: MG01436.1	569 aa
Sequence 2: FG09604.1	635 aa
Sequence 3: NCU07000.1	589 aa
Sequence 4: AN2216.1	541 aa
Alignment Length: 751 aa
Number Identitical Residues: 114 aa
Alignment Score (without introns) 6502


MG01436.1 	-------------------------------------~----------------------
NCU07000.1	-------------------------------------~----------------------
FG09604.1 	MVNRDRPDYGRPDLKSWPRQVRPSARFGFCRIPPSIG1MIISLDDSPGCQRKFGFFRFLT
AN2216.1  	-------------------------------------~----------------------
          	                                                            

MG01436.1 	--~--------------M~DNIVVVNR~NFTSDK0LASGTSCRNYWIDTR~LN-------
NCU07000.1	--~---------------~--------~------~---------------~---------
FG09604.1 	QY1CVAQQGTPIKYPVSV2EFELLQKT0SFSTAK~PDSEVSCRHEISDHS1NANAPSQNN
AN2216.1  	--~---------------~--------~------~---------------~---------
          	                                                            

MG01436.1 	--LAAMIG1SMDAAPLIK0AHDHARNAALAH--TDTTVAINEHALAAGEFSNAARSTTSA
NCU07000.1	--------~-MDLSPLVK0AHDHARAASVATQTADTTVAINHHAQAAGEFANAAKSTTST
FG09604.1 	KLLQSAHG~NMDSSPLVK~AHDHVRAAGVAHQSSDSTVAITEHTQAAGEFANAAKSTSSI
AN2216.1  	--------~-METAPLTL0AHTHARNAALETRKSNPVAASEEHDLAA-------------
          	          *: :**   ** *.* *.:   .::...*  .*  **             

MG01436.1 	EALRTLKLLE~QHHQRLSDLLKLPLERPPRRSQADDYRSGADASHNDDVSEKPSARPASS
NCU07000.1	EALRTLALLE~QHHKRLAELLKVPIEPTSQPSSVDSDIP------EEDEEKASPGQDAGI
FG09604.1 	EALRTLKLLE~EHHRKIADILKRPTAPTP---QVDDT----------DVSEKDSSDKTSI
AN2216.1  	---------E1QHHKKLAHILRFQHENPP------------------------------S
          	         * :**::::.:*:     ..                               

MG01436.1 	GADDKSTADDMSSRRKSAVASGTTSSQQRRYPPSRELSSSIASNLASARGIRSSHRGH--
NCU07000.1	KSAKSDRAGPPSLATPPSAAKPLPTLSQHPRYRQRDLSSSIASKLASARGIRSRYQGQSQ
FG09604.1 	ASQD--AHDKKENKNDSPASKKIAPPTQRRY-AHREMSSSIASNLANARGIPSKYRGQ--
AN2216.1  	STAGVNNQTAAESGASKTDTQQPPKLIENPRLPKRETSS-IASNLASARGIPAQPRHSS-
          	 :   .     .     . :.  .   :.     *: ** ***:**.**** :  :  : 

MG01436.1 	PLAPSVSNDQAPGSLDGHPSRDVGRSKMQN---MLGQKP--------KPSWVPPT-EGPQ
NCU07000.1	PLNPSVSNDQAPGNLEVHPRNREGSLRGGNKGSEVSDRAQK-----QQPSWVPPG-KTSP
FG09604.1 	PLAPSVSNDQAPGNLDAASHSRGTKAKMQN---IIDHRSG-------KPTWVPPA-PVTT
AN2216.1  	PASPTVTSQQAGAKWTEGPAKIRSGESRLRAAQAAGQRDRVNRPLGQKQPWSPPAGSPTD
          	*  *:*:.:** ..    .          . ..  ..:   . . ..: .* ** .  . 

MG01436.1 	KSE---KGQ--ARQQAERPAEESNEDGYSRFYSTFGSLIKGLSAPLAFAGLPLIAEESA-
NCU07000.1	TKED-AAGSSSKPQISGPDSPTATDDGFARFYSTFGSLINRLSAPLAFAGLPLTAEDSSD
FG09604.1 	RSDSHGKGSSSPRSDAASSVVASDDGGYTRFYNAFGSIMNKISAPLAFAGLPLIQEESS-
AN2216.1  	ITSQQFVPAETVDSTQQSYRQSTTEEPFQRFYSTFEGLISKISAPLAFAGLPLGLDSVN-
          	 ...     .:  .        : :  : ***.:* .::. :***********  :.   

MG01436.1 	PEPPTSPDPAATKK-SRPSRAQ-----HVAPEPDLARIYSKATLQALGHKG-----HAPN
NCU07000.1	TASPVLSPEIAPRSGSKPPRQPKATTVSASSDPDLTKIYSRATLRTLARDG-----HAPA
FG09604.1 	ISEPQDSPEMSPKR-SHLKVSP-----SKVPEPDISKIYSQATLKTLAREG-----HGPT
AN2216.1  	NASAVRKSSAETRLDRQTAVSDRS---VSSAEPDISKIFSRAALKAIRESGGGMGTGNPA
          	   .       .: . :      :      .:**:::*:*:*:*::: ..*.. .:  * 

MG01436.1 	DSFYFVPATGGTVSYANILSFDQKERRRNLASIHDGHDHPEMSPED--AADEDDFVDARE
NCU07000.1	DSFYVVPTSGHTASYASILNHEQKEKRRLAASIHGHRDHGNGHPLDDQEEDEDDFVDARE
FG09604.1 	DSFYVVPSTGHTMSYANILSFAEKEKRRLGASSHS--DLLDVLDE-----DDDDFVDARE
AN2216.1  	ESFYVVPTTGGTVSYAGILTRAEKEARR--NSVDD---------------AEDDFVDARE
          	:***.**::* * ***.**.  :** **   * ..                :********

MG01436.1 	SQA------------SSSMSPGVKRRIGSSKSTSDLQNVVEELYLENKGLKEMLDKLSKR
NCU07000.1	SQIQSPPVLPLSSSSAATNPSAFRKRLGRPKTEKDLTNVIEELHMENQTLKDALDKVSRR
FG09604.1 	A---------------PSLSPGAKRRIGRASTDRELNNTIEELYTENKSLKDMLDKLTKR
AN2216.1  	TPSS-----PEMLQASKAKGKGKSGRGNDKLTSLQSPKTLEELQMENQALRHLSDTLSKR
          	:  .     .   .:: :   .   * .   :  :  :.:***  **: *:.  *.:::*

MG01436.1 	LHAFEANAQN--YALQSSMRLVRP--------------GSPLSSSGGGGGGSGGDE----
NCU07000.1	LHTFEVHSQTSTLALAQSIRLQHPGTGTGTGTGGTAGHGSPINASGVMGATAQGGARPEE
FG09604.1 	LHAFEASAQTSAMALAESYRLMRP--------------GSPSASP---HLSKVADE----
AN2216.1  	LHMWEVNAQSSSLALQQSLKAMHHQN-------IPSPEHGPQSVSAVSSPSTGPSTATAA
          	** :*. :*.:: ** .* :  :  .        .:   .*   ..        .  .  

MG01436.1 	--GLRRRNQELEQELAAAVKRMEALEKEYMHVRKVNGQYRERWEKLKAGAKARREAQVAS
NCU07000.1	VAALKRRNRELEEQMMNMTRQMAVMEKDYDKLQKTVEKYRERWEQLKAGAKARREAQQSL
FG09604.1 	--NLRRKNQEIEEQLAAAVKQIERLEKDNRKMQKVLDKYREKWETLKAGAKARREAQGVG
AN2216.1  	PTEQEKRIKELEEALRHHEKELEKVGRENEKLHNVLGRYRDRWEKLKEGAKSRRAEGRGG
          	 :  .:: :*:*: :    :.:  : ::  ::::.  :**::** ** ***:**      

MG01436.1 	GEGESSRSQ-----AG------------------------
NCU07000.1	SGQGSGGNQGEGVGAGEAA---------------------
FG09604.1 	-ESVDDAST-----TG------------------------
AN2216.1  	DSGAKTSSTGTAQQNAGTEGKTGETEQVASNEQSDPKDEA
          	.   .  . . .   . : ..:..:.. ::...:.....:
```
